# Assessing the causal relationships between human blood metabolites and the risk of NAFLD: A comprehensive mendelian randomization study

**DOI:** 10.3389/fgene.2023.1108086

**Published:** 2023-03-28

**Authors:** Ziwei Guo, Tingyu Zhang, Zhangjun Yun, Qian Jin, Xu Cao, Deming Kong, Yuhao Yao, Xiaoke Li, Jiaxin Zhang, Yong’An Ye

**Affiliations:** ^1^ Dongzhimen Hospital, Beijing University of Chinese Medicine, Beijing, China; ^2^ The First Clinical Medical College, Beijing University of Chinese Medicine, Beijing, China; ^3^ Institute of Liver Diseases, Beijing University of Chinese Medicine, Beijing, China

**Keywords:** non-alcoholic fatty liver disease, blood metabolites, mendelian randomization, biliverdin, causality

## Abstract

**Background:** Non-alcoholic fatty liver disease (NAFLD) is a liver disease associated with obesity, insulin resistance, type 2 diabetes mellitus (T2DM), and metabolic syndrome. The risk factors for NAFLD have not been identified. Metabolic dysfunction has been found to be an important factor in the pathogenesis and progression of NAFLD. However, the causal impact of blood metabolites on NAFLD is unclear.

**Methods:** We performed a two-sample Mendelian randomization (MR) study. A genome-wide association study (GWAS) with 7824 participants provided data on 486 human blood metabolites. Outcome information was obtained from a large-scale GWAS meta-analysis of NAFLD, which contained 8,434 cases and 770,180 controls of Europeans. The inverse variance weighted (IVW) model was chosen as the primary two-sample MR analysis approach, followed by sensitivity analyses such as the heterogeneity test, horizontal pleiotropy test, and leave-one-out analysis. In addition, we performed replication, meta-analysis, and metabolic pathway analysis. We further conducted colocalization analysis to deeply reflect the causality.

**Results:** After rigorous genetic variant selection, IVW, sensitivity analysis, replication, and meta-analysis, two known metabolites were identified as being associated with the development of NAFLD [biliverdin: OR = 1.45; 95% *CI* 1.20-1.75; *p* = 0.0001; myristoleate: OR = 0.57; 95% *CI* 0.39-0.83; *p* = 0.0030].

**Conclusion:** By combining genomics with metabolomics, our findings provide a new perspective on the underlying mechanisms of NAFLD and have important implications for the screening and prevention of NAFLD.

## Introduction

Nonalcoholic fatty liver disease (NAFLD) is a clinicopathological syndrome characterized by excessive fat deposition in hepatocytes except for alcohol and other clear liver damage factors, closely related to insulin resistance and genetic susceptibility. It is also the most common cause of chronic liver disease, with a global prevalence of 25%. (Younossi et al., 2016; Cheung et al., 2019; Harrison et al., 2021). NAFLD can progress to non-alcoholic steatohepatitis (NASH) (Barb et al., 2016; Yin et al., 2023) and even hepatocellular carcinoma (HCC) (Feldstein et al., 2009). The prevalence of NAFLD is increasing yearly, but less than 5% of people with NAFLD are aware of their disease status (Alqahtani et al., 2021). No recognized and reliable drug therapies exist, posing a substantial global public health challenge (Younossi et al., 2019; Paternostro and Trauner, 2022). Early recognition and prevention of NAFLD are, therefore, significant.

Diagnosing NAFLD requires costly imaging and invasive procedures, which impose a significant socioeconomic burden (Dorairaj et al., 2021). Patients with NAFLD often have a combination of type 2 diabetes mellitus (T2DM) (Tilg et al., 2017; Targher et al., 2021), metabolic syndrome (MS) (Tapper and Loomba, 2018), and cardiovascular events (Mantovani et al., 2022; Muzurović et al., 2022), with relatively significant changes in their blood metabolomics. However, as there are no susceptible and specific tests to diagnose NAFLD and differentiate NASH from pure steatosis16, there is a lack of reliable biomarkers to assess the progression of NAFLD (Masoodi et al., 2021). Therefore, further relevant studies (e.g., blood metabolomics) are needed to identify biological markers associated with NAFLD, which can provide a basis for diagnosing and treating NAFLD (Piazzolla and Mangia, 2020; Dorairaj et al., 2021).

NAFLD has a complex and multifaceted biochemical metabolic process. In recent years, more studies have suggested that blood biomarkers of NAFLD that are not regulated by secondary non-causal pathways may be promising candidates for identifying individuals at risk (Gobeil et al., 2022). The National Institutes of Health Medical Library (NIH) shows that 228 of the current 1,230 studies on NAFLD are related to blood metabolism. These studies suggested that certain metabolites are involved in the progression of NAFLD. Some guidelines (Vos et al., 2017; Cusi et al., 2022) demonstrate that blood transaminase levels, gamma-glutamyl transferase, serum triglycerides, and a 4-factor-based fibrosis index (FIB-4) can be applied to aid in the assessment and diagnosis of NAFLD but continue to lack specificity. In a study of NAFLD patients who fasted overnight (Kalhan et al., 2011), significantly elevated levels of glycocholate, taurocholate and glycoglycolate were found in NAFLD participants. Masoodi et al. (2021) found changes in circulating fatty acids, triglycerides, phospholipids, and bile acids in NAFLD patients; Gorden et al. (2015) used linear discriminant analysis of a set of 20 plasma metabolites (including glycerophospholipids, sphingolipids, etc.) that can be used to differentiate NASH from simple steatosis offers the potential to improve the clinical diagnosis of NAFLD and facilitate therapeutic interventions. In addition, Gobeil et al. (2022) aimed to identify novel biomarkers of NAFLD through Mendelian randomization. This analysis suggests that a potential causal relationship was revealed between tyrosine levels and NAFLD in a positive manner, which may represent a novel clinical biomarker for NAFLD. However, systematic studies to assess the causal relationship between blood metabolites and NAFLD are still lacking and translating these metabolic findings into pathophysiological mechanisms and new therapies is an enormous challenge. Therefore, a more comprehensive analysis of the interactions between genetics and blood-circulating metabolites in the pathogenesis of NAFLD is still needed.

Mendelian randomization (MR) is a recent and widely used method for epidemiological investigations in which single nucleotide polymorphisms (SNPs) are pooled to infer the causal effect of exposure factors on outcomes (Emdin et al., 2017; Zheng et al., 2017; Hemani et al., 2018). MR uses a genetic variation to simulate the design of randomized controlled trials (RCTs), and genome-wide association studies (GWASs) can be used for flexible two-sample MR analysis. In the case of high cost, time-consuming, and even low feasibility of RCT trials, MR can be used as an alternative to RCT because it relies on the natural random classification of genetic variation during meiosis to generate a random distribution of genetic variation in the population (Richmond and Davey Smith, 2022). It provides reliable evidence for the causal relationship between phenotypes (Zuccolo and Holmes, 2017). MR can also be used to identify biomarkers of disease-related characteristics by determining whether genetic susceptibility to certain diseases affects other biological characteristics, such as blood metabolomics (Mohammadi-Shemirani et al., 2019; Ritchie et al., 2021).

Some MR studies have been performed to explore the relationship between exposure and NAFLD. However, the main focus was single exposures or common exposure factors, such as interleukin-6 (IL-6) (Li et al., 2022a), seven sleep characteristics (Fan et al., 2022), serum uric acid levels (Li et al., 2022b), iron status (He et al., 2022), and coronary artery disease (Ren et al., 2022). Few studies have focused on blood metabolites and NAFLD. Given that the causal relationship between blood metabolites and NAFLD is not well understood, this study used a two-sample MR approach to assess the causal relationship between 486 human blood metabolites and the risk of NAFLD to provide a deeper understanding of the pathogenesis of NAFLD.

## Materials and methods

### Study design

The public dataset, accessible to the public on the database website and has already gained ethical approval, contains all the data we used for this investigation.

**FIGURE 1:**
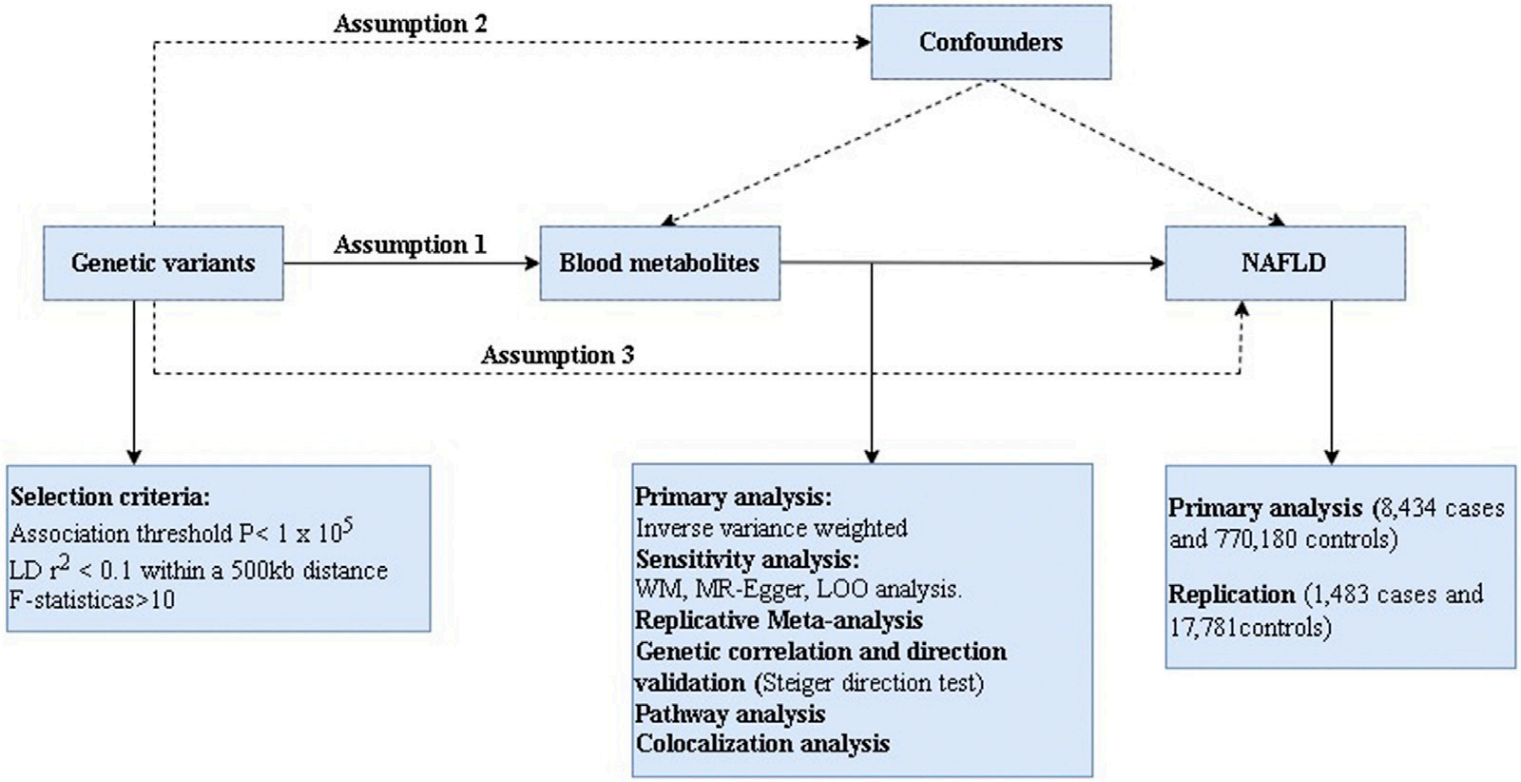
The overview of the research workflow.

In this present work, we comprehensively evaluated the 486 serum metabolites in relation to risk of NAFLD based on a rigorous MR design. A scientific MR study should comply with three major hypotheses: 1) The genetic instruments are strongly correlated with exposures of interest; 2) The genetic variation must be independent of any confounding factors associated with outcome; 3) The genetic instruments can only affect the outcome *via* the exposure. If the genetic instruments affect the outcome *via* other risk factors, it is known as horizontal pleiotropy (Chen et al., 2022). Briefly, we performed MR analysis using GWAS data for 486 blood metabolites (exposure) and NAFLD (outcome) from European population. Notably, considering that the estimates of MR study are affected by the sample size, we obtained two types GWAS data for NAFLD, one for the main analysis and the other for the replication analysis to improve the confidence of the estimates. The overview of this study is shown in Figure 1.

### GWAS data for human blood metabolites

We downloaded summary type GWAS data for human serum metabolites from the Metabolomics GWAS Server (http://metabolomics.helmholtz-muenchen.de/gwas/). Notably, this is the most comprehensive GWAS data to date on blood metabolites which were discovered by Shin et al. (2014) in 2014 from 7,824 European descents. In detail, 2.1 million SNPs for 309 known and 177 unknown metabolites were identified, respectively. According to the Kyoto Encyclopedia of Genes and Genomes (KEGG) database, the 309 known metabolites can be classified into 8 classes: cofactors and vitamins, energy, amino acid, carbohydrate, lipid, nucleotide, peptide, and xenobiotic metabolism (Supplementary Table S1).

### GWAS data for NAFLD

GWAS data for NFALD from a genome-wide meta-analysis based on 4 European cohorts containing 8,434 cases and 770,180 controls were used for the primary analysis (Ghodsian et al., 2021). The diagnosis of NAFLD in these 4 cohorts was determined based on the electronic health records of all participants. We download these GWAS data from the GWAS catalog (https://www.ebi.ac.uk/gwas/) and their GWAS Catalog accession number is GCST90011885. More detailed documentation of this GWAS data can be obtained from the original literature.

### Selection of instrumental variables (IVs)

We developed a series of criteria to screen IVs associated with blood metabolites. Firstly, we set the significance threshold at 1.00E-5 (*p* < 1 × 10^−5^) and the linkage disequilibrium (LD) r^2^ < 0.1 within 500 KB. Considering the limited number of SNPs, we relaxed the association threshold which was widely used in previous MR studies (Cai et al., 2022a; Cai et al., 2022b). Secondly, in order to obtain excellent IVs, the F statistic for each SNP was calculated as previously described (Cai et al., 2022a). In general, the F-statistic <10 is considered weak IVs and discarded (Pierce et al., 2011; Burgess et al., 2013; Bowden et al., 2016b). Thirdly, we extracted the SNPs for exposure of interest from the outcome and excluded those related to the outcome (*p* < 1 × 10^−5^). Then we conducted harmonization to align the exposure- and outcome-SNPs alleles and discard palindromic SNPs with intermediate effect allele frequencies (EAF > 0.42) or SNPs with incompatible alleles. Finally, these retained metabolites were used for MR analysis (Gill et al., 2019).

### Primary analysis

Given that the random-effect inverse variance weighted (IVW) provided the most precise estimates under the premise that all SNPs were valid. We used IVW method as the primary analysis to asses causality between blood metabolites and NAFLD with *p* < 0.05. The IVW method is ideal for estimating robust causal detection ability (Pierce and Burgess, 2013). It was proposed by Burgess et al. (2013), and was widely used for MR studies.

### Sensitivity analysis

To enhance the confidence of the estimates, we used Weighted median (WM) and MR-Egger as complementary analyses because they possess strengths under different premises. The WM method provides consistent causal estimates when >50% of the weight comes from valid instruments (Bowden et al., 2016a), while MR-Egger regression accounting for pleiotropy when all the instruments are invalid (Bowden et al., 2015). For sensitivity analysis, we used four analysis methods including Cochran-Q test, MR-Egger intercept, leave-one-out analysis (LOO) and MR-PRESSO. Cochran-Q derived *p* < 0.05 and I^2^>25% was considered as existing heterogeneity (Greco et al., 2015). And horizontal pleiotropy was evaluated based on MR-Egger intercept (Bowden et al., 2016b). To determine whether the MR estimates was influence by a single SNP, a LOO analysis was also conducted (Burgess and Thompson, 2017).

As a result, the following criteria were used to identify the likely suitable candidate metabolites implicated in the development of NAFLD: 1) Uniformity of magnitude and directions across the 4 MR techniques; 2) No pleiotropy or heterogeneity was found; 3) No LOO analysis revealed any strong impact sites.

### Replication and meta-analysis, and metabolic pathway analysis

For the estimates of significant associations (*P*_IVW_ < 0.05), replication analysis and meta-analysis were implemented to determine the final candidates through additional GWAS data for NAFLD from the GWAS Catalog which GWAS Catalog accession number is GCST90091033 including 1,483 cases and 17,781 controls. We based the metabolic pathway analysis on the KEGG database using MetaboAnalyst 5.0 (https://www.metaboanalyst.ca/) for metabolic pathway analysis of known metabolites.

### Genetic correlation and direction validation

Previous studies have suggested that MR results may have false positives due to genetic correlations between traits (O'Connor and Price, 2018). Throughout the instrument selection process, SNPs associated with NAFLD were removed, and combinations of SNPs not significantly associated with NAFLD may also contribute to the genetic risk of NAFLD. Thus, the genetic relationship between the identified metabolites and NAFLD was evaluated by linkage disequilibrium score regression (LDSC) to ascertain whether the causal effects were disturbed by shared genetic architecture. Additionally, we used the Steiger test to confirm if the observed causalities were biased due to reversed causation (Hemani et al., 2017). This test determined whether the included SNPs explained more about NAFLD variability than the detected metabolites. When a combination of SNPs was found to have no genetic risk for NAFLD compared to metabolites, the results indicated no bias in causal inference (Steiger *p* < 0.05).

### Colocalization analysis

Colocalization analysis was applied to detect whether the exposure and outcome share a common causal variant in a given region (Wang et al., 2021a). Colocalization analysis is now a standard part of MR analysis, and it is increasingly common to conduct MR analysis in conjunction with corresponding colocalization analysis. MR differs from colocalization analysis in that MR analysis prioritises evidence of causality, whereas colocalization analysis is more conservative and can be an important complementary analysis to support MR analysis in assessing the validity of instrumental variable hypotheses (Gaziano et al., 2021). Based on this, colocalization analysis methods such as expression quantitative trait loci (eQTLs) and protein quantitative trait loci (pQTLs) were developed. The principle is to use eQTL and pQTL loci published in existing databases, combined with GWAS summary data, to identify eQTL and pQTL loci associated with phenotypes (Sun et al., 2018). For statistically significant MR results, we also performed colocalization analysis using the moloc R package (https://github.com/clagiamba/moloc).

### Statistical analysis

All MR analyses were performed using the “TwoSampleMR” package (version 0.4.22). The meta-analysis was performed by the Reviewer Manager software (Version 5.4.1) and LDSC was conducted by LDSC software (version 1.0.1), *p* < 0.05 was considered statistically significant. We used the odds ratio (OR) as the main effect indicator along with its corresponding 95% confidence interval (CI).

## Results

Following the strict instrument selection steps, we performed MR analyses on 486 blood metabolites. Five of the 486 blood metabolites appeared in two forms, totaling 491 MR analyses. F statistics were all greater than the empirical threshold 10, with a minimum of 17, suggesting that all SNPs had sufficient validity. The F statistic for all SNPs was shown in Supplementary Table S2.

### Primary analysis and sensitivity analysis

**FIGURE 2:**
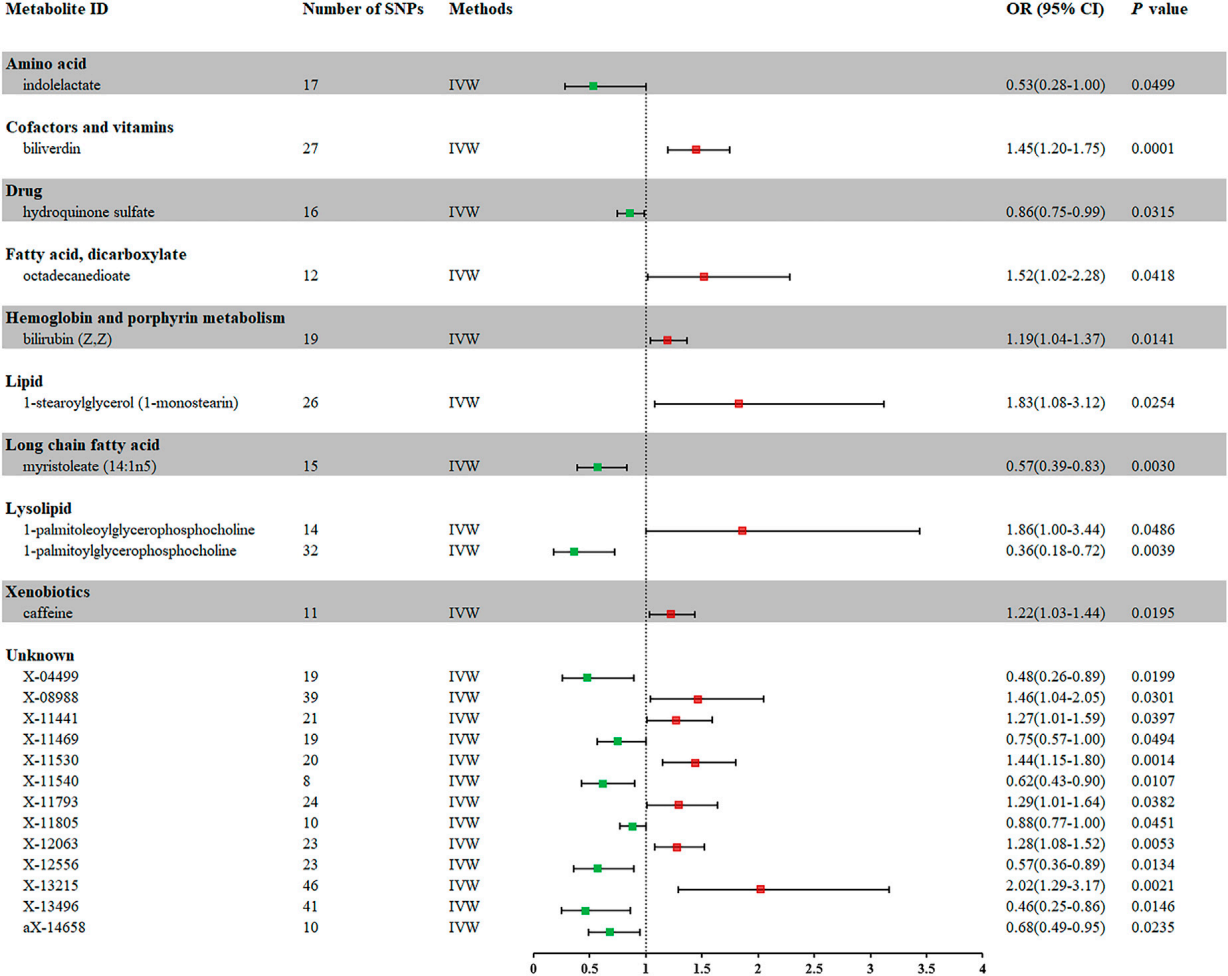
Forest plot for the causal effect of metabolites on the risk of NAFLD derived from IVW. OR, odds ratio; *CI*, confidence interval.

**FIGURE 3:**
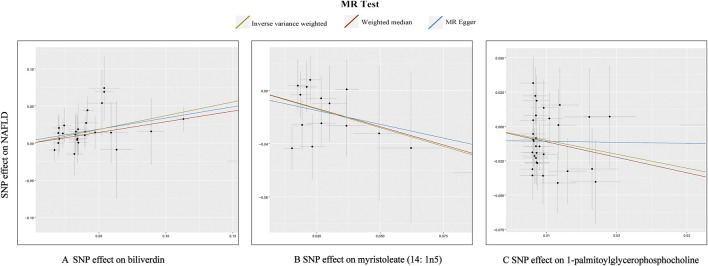
Scatterplot for the significant MR association (*p* < 0.05) between metabolites and NAFLD. **(A)**: biliverdin; **(B)**: myristoleate; **(C)**:1-palmitoylglycerophosphocholine.

In total, 23 metabolites were preliminarily identified by IVW as significantly linked with NAFLD (Figure 2; Supplementary Tables S3, S4). 13 of them still have unidentified chemical compositions. Additional ten metabolites were chemically categorized as belonging to the metabolism of amino acids, cofactors, vitamins, lipids, drugs, fatty acids, dicarboxylates, hemoglobin, porphyrins, long-chain fatty acids, lysolipids, and xenobiotics. After com supplementary analysis and sensitivity analysis, only three metabolites met the criteria of eligible candidate metabolites in relation to risk of NAFLD, including biliverdin (odds ratio (OR) = 1.45; 95% confidence interval (95% *CI*) 1.20-1.75; *p* = 0.0001), myristoleate (OR = 0.57; 95% *CI* 0.39-0.83; *p* = 0.0030) and 1-palmitoylglycerophosphocholine (OR = 0.36; 95% CI 0.18-0.72; *p* = 0.0039) (Figure 3). Specifically, the robustness of the causation was supported by MR estimates produced from WM and MR-Egger that showed consistent direction and magnitude. Cochran Q-derived *p* values indicated that no heterogeneity was detected. Besides, intercept from MR-Egger suggested no horizontal pleiotropy (Table 1). Additionally, LOO analysis failed to find any high-influence SNPs that would have biased the pooled effect estimates (Supplementary Figure S1A–C). It was concluded that these three metabolites should be further investigated as potential candidate metabolites involved in the pathogenesis of NAFLD. As the threshold of *p*-value is artificially specified, no matter how small the *p*-value is, it only represents a low false positive result and does not guarantee a true result. Furthermore, *p* < 0.05 is a very lenient threshold and we need to perform multiple testing to achieve the elimination of false positives by correcting the threshold for the *p*-value. The formula for the Bonferroni correction is p*(1/486), where p is the original threshold and 486 is the total number of tests. After the Bonferroni correction, only bilirubin passed this criterion; all other metabolites were nominally significant.

**TABLE 1:** Three MR models estimate the causal relationships between 10 known metabolites and the risk of NAFLD and tests for heterogeneity and horizontal pleiotropy. WM, weighted median; IVW, inverse variance weighted.

Metabolite	Methods	SNP N)	OR (95% *CI*)	*P*	Heterogeneity	*P*	Pleiotropy	*P*
					Q value (I^2^)		Intercept	
caffeine	MR Egger	11	1.19 (0.61–2.33)	0.6198				
	WM	11	1.21 (0.97–1.50)	0.0927				
	IVW	11	1.22 (1.03–1.44)	0.0195	5.34	0.87	0.00	0.95
biliverdin	MR Egger	27	1.35 (0.95–1.92)	0.1047				
	WM	27	1.33 (1.01–1.76)	0.0434				
	IVW	27	1.45 (1.20–1.75)	0.0001	14.31	0.97	0.00	0.66
indolelactate	MR Egger	17	0.69 (0.17–2.79)	0.6064				
	WM	17	0.66 (0.29–1.48)	0.3126				
	IVW	17	0.53 (0.28–1.00)	0.0499	23.86	0.09	−0.01	0.69
1-stearoylglycerol (1-monostearin)	MR Egger	26	0.43 (0.11–1.64)	0.2262				
	WM	26	1.06 (0.50–2.22)	0.8827				
	IVW	26	1.83 (1.08–3.12)	0.0254	33.11	0.13	0.03	0.03
bilirubin (Z, Z)	MR Egger	19	1.32 (1.00–1.74)	0.0706				
	WM	19	1.21 (1.01–1.46)	0.0408				
	IVW	19	1.19 (1.04–1.37)	0.0141	19.85	0.34	−0.01	0.44
myristoleate (14:1n5)	MR Egger	15	0.66 (0.30–1.47)	0.3287				
	WM	15	0.58 (0.34–0.97)	0.0374				
	IVW	15	0.57 (0.39–0.83)	0.0030	10.92	0.69	0.00	0.69
1-palmitoleoylglycerophosphocholine*	MR Egger	14	3.30 (0.43–25.41)	0.2733				
	WM	14	1.03 (0.46–2.32)	0.9390				
	IVW	14	1.86 (1.00–3.44)	0.0486	14.15	0.36	−0.01	0.57
1-palmitoylglycerophosphocholine	MR Egger	32	0.93 (0.12–7.10)	0.9423				
	WM	32	0.32 (0.12–0.91)	0.0316				
	IVW	32	0.36 (0.18–0.72)	0.0039	28.85	0.58	−0.01	0.34
hydroquinone sulfate	MR Egger	16	0.80 (0.61–1.04)	0.1202				
	WM	16	0.82 (0.68–0.98)	0.0294				
	IVW	16	0.86 (0.75–0.99)	0.0315	8.42	0.91	0.01	0.51
octadecanedioate	MR Egger	12	1.76 (0.72–4.30)	0.2400				
	WM	12	1.20 (0.71–2.03)	0.4853				
	IVW	12	1.52 (1.02–2.28)	0.0418	6.01	0.87	−0.01	0.72

The univariate MR analyses provided persuasive evidence for a causal relationship between blood metabolites and NAFLD. To confirm the actual association between blood metabolites and NAFLD, we performed a multivariate MR (MVMR) analysis. MVMR analysis assesses the direct effect of the exposure of interest on the outcome by controlling for potential effects between exposures. In this study, MVMR analysis was performed based on multiplicative inverse variance weighting of multivariate random effects. Our MVMR analysis can provide evidence that the three metabolites are independent of each other and can directly affect NAFLD independent of the other metabolites (Supplementary Table S5).

### Replication and meta-analysis

**FIGURE 4:**
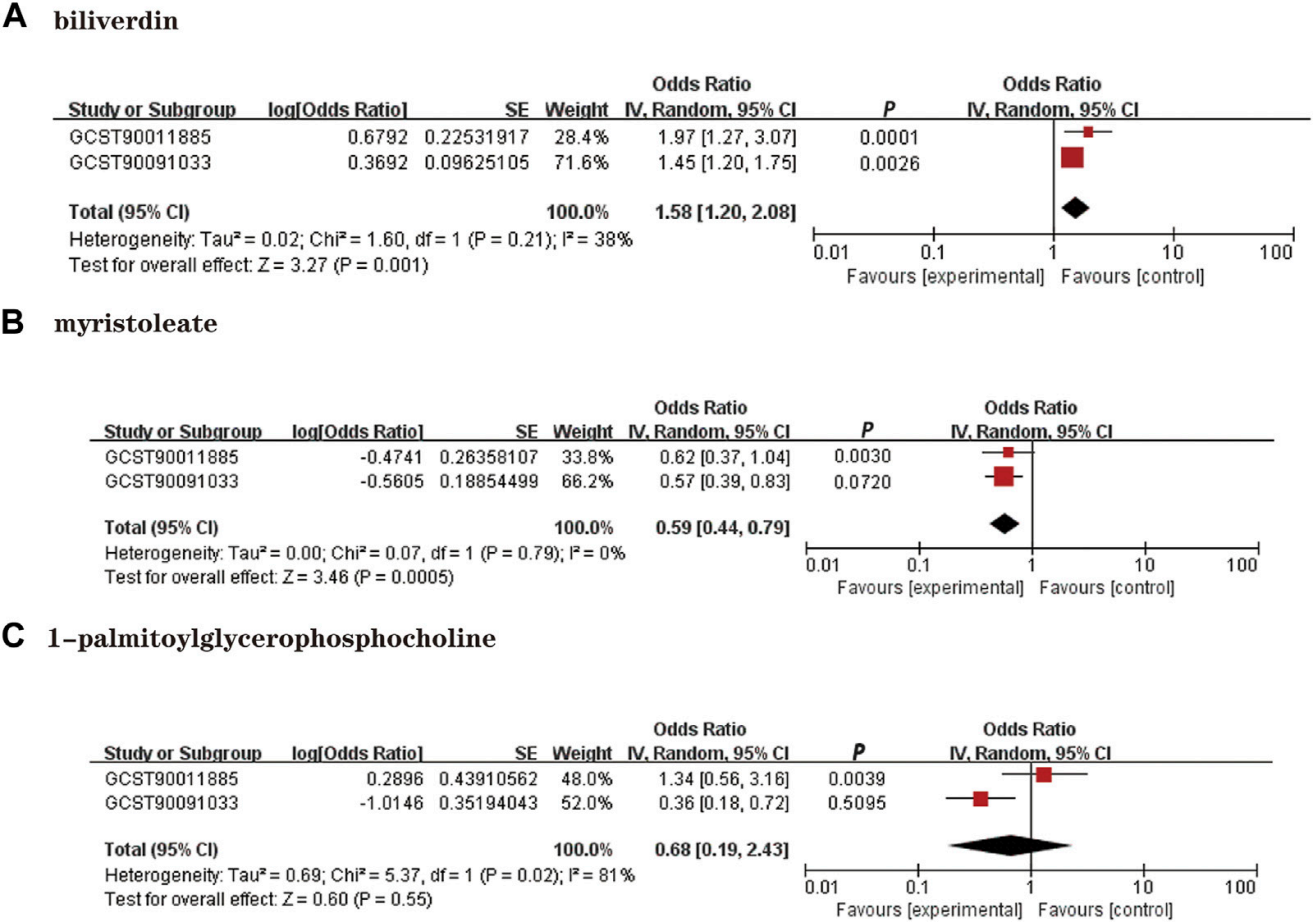
Meta-analysis of the causal associations between metabolites and NAFLD. GCST90011885: Primary analysis of NAFLD GWAS; GCST90091033; OR: Replication analysis of NAFLD GWAS. **(A)**: Biliverdin; **(B)**: myristoleate; **(C)**:1-palmitoylglycerophosphocholine.

We performed a replication analysis using another NAFLD GWAS data to validate our results further. A meta-analysis of 3 known metabolites with stable MR results was performed in combination with 2 GWAS datasets, and the results were as expected (Figure 4), with high levels of genetic predisposition to biliverdin (OR = 1.58; 95% *CI* 1.20-2.08; *p* = 0.001) predicting increased risk of NAFLD and higher levels of gene susceptibility to myristoleate (OR = 0.59; 95% *CI* 0.44-0.79; *p* = 0.0005) predicted a lower risk of NAFLD. However, the meta-analysis results were not observed to be statistically significant in 1-palmitoylglycerophosphocholine (*p* = 0.55).

### Genetic correlation and direction validation

The results of LDSC showed weak evidence that genetic correlation between NAFLD and biliverdin (r_g_ = 0.2204, se = 0.3922, *p* = 0.5742), myristoleate (r_g_ = 0.1247, se = 0.2965, *p* = 0.6739), and 1-palmitoylglycerophosphocholine (r_g_ = 0.0580, se = 0.2859, *p* = 0.8393), suggesting that the shared genetic component did not confound the MR estimates (Supplementary Table S6). Furthermore, we further performed the Steger-test to examine whether there was reverse causality between metabolites and NAFLD. The results of Steiger do not support the existence of reverse causal effects between metabolites and NAFLD (*p <* 0.05) (Table 2).

**TABLE 2:** Steiger direction test from blood metabolites to NAFLD.

Exposure	biliverdin	myristoleate	1-palmitoylglycerophosphocholine
Direction	TRUE	TRUE	TRUE
Steiger P	0.00E+00	3.02E-82	4.92E-156

### Metabolic pathway analysis

We input ten known metabolites into Metabolic Analyzer 5.0 to determine various potential metabolic pathways involved in the pathogenesis of NAFLD (Table 3). Among them, biliverdin and bilirubin were involved in the metabolic pathways of porphyrin and chlorophyll metabolism, and coffee was involved in the caffeine metabolism pathway (*p* < 0.05). The metabolic mechanism formed by the above metabolites may be involved in the pathogenesis and development of NAFLD.

**TABLE 3:** Signifcant metabolic pathways involved in the pathogenesis of NAFLD.

Pathway Name	Involved metabolites	*P*
Porphyrin and chlorophyll metabolism	Biliverdin and Bilirubin	0.0011
Caffeine metabolism	Caffeine	0.0192

### Colocalization analysis

For biliverdin with significant results, we performed a colocalization analysis of NAFLD risk using the coloc R package. BLVRAD eQTL files from the eQTLGen Consortium (https://www.eqtlgen.org/index.html). The eQTLGen Consortium has been set up to identify the downstream consequences of trait-related genetic variants. The consortium incorporates 37 datasets, with a total of 31,684 individuals. Colocalization analysis to further determine the probability of shared causal genetic variation in SNP associated with NAFLD and eQTL.In this study, only cis-eQTL were included to generate genetic tools, i.e., eQTL encoding genes within 1 Mb on either side of the gene. For NAFLD, we extracted the region upstream and downstream of the BLVRAD significant locus (plus or minus 1024 kb, r^2^ < 0.2) from NAFLD GWAS data as colocalization region 1. The results showed that GWAS signals and eQTL colocalization were not detected and that BLVRAD and NFALD did not share a causal variant (H4 = 0.0122) (Supplementary Table S7).

Protein expression data for BLVRAD were obtained from the deCODE Consortium (https://www.decode.com/summarydata/). Colocalization analysis can further determine the probability of shared causal genetic variation in snp associated with NFALD and pQTL. Significant colocalization (posterior probability) was set to PP.H4 > 0.95, i.e., genes strongly colocalised with NAFLD were considered as potential target molecules. The results found strong evidence that BLVRAD in blood may be a potential target molecule for NAFLD (PP.H4 = 0.9579) (Supplementary Table S8). Therefore, associations between colocalization at the protein level and DNA sequence variants with NAFLD risk allele level could further explore the mechanisms of the disease and reveal novel drug targets and biomarkers.

## Discussion

**FIGURE 5:**
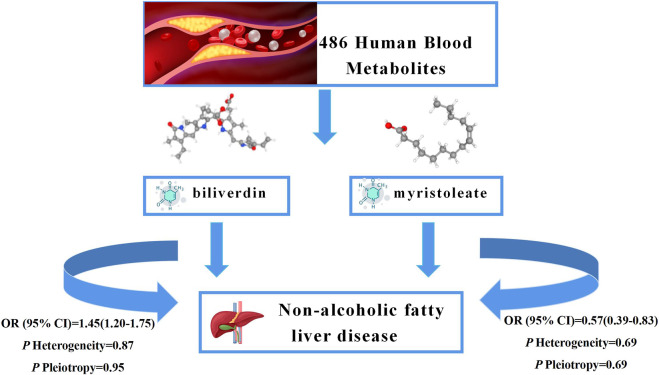
Graphical summary. Among 486 human blood metabolites, this study found that the higher levels of biliverdin (OR = 1.45; 95% *CI* 1.20-1.75; *p* = 0.0001) are causally associated with an increased risk of NAFLD and the higher levels of myristoleate (OR = 0.57; 95% *CI* 0.39-0.83; *p* = 0.0030) play a protective role in the development of NAFLD.

As NAFLD is a metabolic stress liver injury closely related to genetic susceptibility, the influence of genetic factors on hepatic steatosis has been reported in recent experimental and observational studies (Younossi et al., 2016; Martin et al., 2021; Oliveira et al., 2021). In this study, we performed an unbiased two-sample MR analysis to causally assess 486 blood metabolites and the risk of NAFLD. We collected the most extensive mGWAS and large NAFLD GWAS summary data from public databases. We performed an initial IVW analysis of 486 metabolites using genetic variants as IVs and ultimately identified a causal relationship between 23 metabolites and NAFLD, 10 known metabolites. We then performed heterogeneity tests and sensitivity analyses on these metabolites. In addition, to further ensure the reliability and stability of the results, we used other databases for validation and performed a meta-analysis and metabolic pathway analysis. The results suggest that higher levels of biliverdin (OR = 1.45; 95% *CI* 1.20-1.75; *p* = 0.0001) are causally associated with an increased risk of NAFLD and that higher levels of myristoleate (OR = 0.57; 95% *CI* 0.39-0.83; *p* = 0.0030) play a protective role in the development of NAFLD. The graphical summary of this study shows in the Figure 5. To our knowledge, this is the first MR study to assess the causal role of human blood metabolites systematically and comprehensively in NAFLD.

The prevalence of NAFLD and the lack of pharmacological therapies place a heavy burden on the world, making the screening and prevention of NAFLD particularly important. Although guidelines (Cusi et al., 2022) have mentioned several complementary diagnostic indicators for NAFLD (ALT, GGT, etc.), there is still a lack of susceptible and specific diagnostic indicators for NAFLD and biomarkers to assess the progression of NAFLD. Given that the gold standard for diagnosing NAFLD remains liver biopsy, which is costly and invasive, further research is needed to find specific diagnostic indicators for NAFLD. Previous studies (Saeed et al., 2017; Zhao et al., 2020; Notarnicola et al., 2021) have identified various blood metabolite changes in NAFLD patients, such as imbalances in triglyceride metabolism, disturbances in vitamin A metabolism, and elevated plasma N-trimethyl-5-aminovaleric acid (TMAVA) levels in mice with hepatic steatosis. Despite the increasing number of studies on the blood metabolism of NAFLD, there is still a lack of comprehensive and systematic studies to assess the causal relationship between NAFLD and blood metabolites. Inspired by the work of Cai et al. (2022b), Yu et al. (2022), and Wang et al. (2021b) in exploring the causal relationship between metabolites and disease, we designed this MR analysis of NAFLD and blood metabolites to assess the causal relationship between the two systematically.

Our findings suggest that high biliverdin levels increase the risk of NAFLD progression, but there is a paucity of research on the direct correlation between biliverdin and NAFLD. Bilirubin is a by-product of hemoglobin catabolism. Hemoglobin is degraded by heme oxygenase-1 (HO-1) to biliverdin, which is rapidly converted to bilirubin by the action of biliverdin reductase A. Bilirubin and biliverdin reductase A (BVRA) have been found to protect the liver from lipid accumulation as well as disease (Lin et al., 2009; Kwak et al., 2012; Puri et al., 2013). Hepatic BVRA inhibits glycogen synthase kinase-3β by enhancing serine nine phosphorylation, thereby preventing hepatic steatosis (Hinds et al., 2016). After oxidation of bilirubin to biliverdin in the mitochondria, biliverdin must be exported to the cytoplasmic lysate for reduced bilirubin, and studies have found enhanced redox of bilirubin increases insulin resistance and steatosis in obese patients (Shum et al., 2021). From this, we can speculate that the increase in biliverdin levels indirectly reflects an enhanced bilirubin redox process, which affects the progression of NAFLD. However, a related study (Ikeda et al., 2011) found that biliverdin prevented the deterioration of abnormal glucose tolerance in mice with T2DM. NAFLD is commonly associated with metabolic risk factors such as obesity, dyslipidemia, hypertension, and diabetes, and the global rise in the prevalence of obesity and type 2 diabetes has coincided with an increase in the prevalence of NAFLD (Loomba et al., 2021); Biliverdin has antioxidant and anti-inflammatory properties (Shiels et al., 2020), and the progression of NAFLD can be affected by inflammation and oxidative stress damage, although the results of these studies may be limited by methodological flaws such as residual confounding and other factors. Colocalization analysis has been proven a powerful tool in revealing the pleiotropic effects of certain loci on multiple traits (Wang et al., 2021a). Proteins are more likely to be used as drug targets than other molecular traits and MR analysis combined with the use of pQTL as colocalization for IV will be valuable to the wider community of human genetics. The results of pQTL in this study suggest that BLVRAD is a protein with high supporting colocalization evidence (PP.H4 = 0.9579), and this association provides a reference for further exploration of the pathogenesis of NAFLD and revealing novel drug targets and biomarkers. Although the colocalisation results of eQLT are negative, it does not necessarily mean that the study is meaningless.

Up to now, research on the relevance of myristoleate to NAFLD is minimal. One study has shown that myristoleate is expressed at high levels during mid-development in oyster larvae and is one of the metabolites associated with fatty acid metabolism (Liu et al., 2020). A related study found that myristoleic acid produced by *E. faecalis* reduced obesity through brown fat activation and beige fat formation (Quan et al., 2020). In contrast, a study found that fatty acids (myristoleic acid) during adipogenesis were associated with an increased risk of T2DM (Qureshi et al., 2019). Since obesity and T2DM are risk factors for the development of NAFLD, we hypothesize that myristoleate, which is involved in fatty acid metabolism, may play a role in controlling the progression of NAFLD. And through our MR analysis study, we found that genetic predisposition towards higher levels of myristoleate played a protective role in NAFLD development and can inhibit the progression of NAFLD. However, experimental studies on NAFLD and myristoleate are lacking. Therefore, the protective mechanism of myristoleate needs to be further explored.

In addition, the association of caffeine with the risk of NAFLD remains highly controversial, and although nearly 41% of studies have concluded that caffeine is protective against NAFLD, there are also studies showing that caffeine increases hepatocyte damage in mice and definitive research evidence that prenatal caffeine exposure increases susceptibility to NAFLD in rat offspring (He et al., 2019; Hu et al., 2019; Dungubat et al., 2020). Therefore, our genome-wide association study finding that caffeine may be a risk factor for NAFLD is not contrary to the facts and may be related to the sample size and sample population involved in this study. This suggests that the association between caffeine and NAFLD needs to be examined in a more thorough and comprehensive study.

Our study has certain advantages. Firstly, from the perspective of molecular mechanism, it has a solid theoretical basis and important clinical research value to explore the causal relationship between metabolites and NAFLD by using blood metabolites as exposure factors. Secondly, this study used strict quality control conditions and rational, analytical methods, including various models, to evaluate causal effects, which largely avoided reverse causality and residual confounding. Also, the use of large-scale GWAS data gives it greater statistical validity. In addition, statistical methods such as meta-analysis and Stegall’s test (Hemani et al., 2017) were used to test the validity of the MR results. Therefore, the results of this study are mainly reliable and stable. Thirdly, unlike previous MR analyses of single or conventional exposure factors, analyzing 486 blood metabolites is a more difficult task and presents statistical analysis challenges. The analytical strategy we propose may be informative for similar studies in the future. However, several limitations should be noted in our study. First, all mGWAS and NAFLD GWAS data were from European populations. Although this largely avoids population heterogeneity, the MR results should be further validated in other populations to verify their generalisability in future studies with more GWAS data. Second, more than half of the NAFLD risk predictors obtained through preliminary analyses were unknown metabolites whose functional structures are unknown. Therefore, the results of this study are limited. Third, we revealed that cholestyramine and methyl myristate are nominally causally related to NAFLD using a two-sample MR approach. However, this relationship is theoretical, and we failed to confirm it mechanistically. Therefore, the results of the MR study should be further validated in a robust RCT to demonstrate the existence of a causal relationship.

In conclusion, we have identified a causal relationship between two blood metabolites and NAFLD by MR analysis, providing preliminary evidence of the effect of these two metabolites on the progression of NAFLD. This may help establish individualized explanations or markers for biological differences in disease states and serve as candidate molecules for future mechanistic exploration. However, due to the limited experimental studies on biliverdin, myristoleate, and NAFLD, the mechanisms by which the two metabolites affect NAFLD progression are unclear. Therefore, more studies may be needed to explore whether these two metabolites can be used as clinical circulating biomarkers for the screening and prevention of NAFLD.

## Data Availability

The datasets presented in this study can be found in online repositories. The names of the repository/repositories and accession number(s) can be found in the article/Supplementary Material.

## Abbreviations

CI, Confidence interval; eQTLs, Expression quantitative trait loci; EAF, Effect allele frequencies; GWAS, Genome-wide association studies; HCC, Hepatocellular carcinoma; IVW, Inverse variance weighted;IL-6, Interleukin-6; Ivs, Instrumental variables; KEGG, Kyoto Encyclopedia of Genes and Genomes; LD, Linkage disequilibrium; LOO, Leave-one-out; MS, Metabolic syndrome; MR, Mendelian randomization; MVMR, Multivariate MR analysis; NAFLD, Non-alcoholic fatty liver disease; NASH, Non-alcoholic steatohepatitis; OR, Odds ratio; pQTLs, Protein quantitative trait loci; RCT, Randomized controlled trials; SNP, Single nucleotide polymorphisms; T2DM, Type 2 diabetes mellitus; WM, Weighted median.
