# Supplementary material for: Assessing the causal relationships between human blood metabolites and the risk of NAFLD: A comprehensive mendelian randomization study
Source: Front Genet. 2023 Mar 28;14:1108086. doi: 10.3389/fgene.2023.1108086 (PMC10086196; doi:10.3389/fgene.2023.1108086)

***Supplementary Material***

**Assessing the causal relationships between human blood metabolites and the risk of NAFLD: A comprehensive Mendelian randomization study**

**Ziwei Guo ^1,2†^, Tingyu Zhang ^1,2^, Zhangjun Yun ^1,2^, Xu Cao ^1,2^, Deming Kong ^1,2^, Yuhao Yao ^1,2^, Xiaoke Li ^1, 3*^,Jiaxin Zhang ^1, 3*^and** **Yong’an Ye ^1, 3*^**

*** Correspondence:**Yong’an [Ye,yeyongan@vip.163.com](mailto:Ye,yeyongan@vip.163.com),Jiaxin Zhang,

[happyjiaxin@bucm.edu.cn](mailto:happyjiaxin@bucm.edu.cn) and Xiaoke Li, [lixiaoke@vip.163.com](mailto:lixiaoke@vip.163.com).

**Supplementary Figures**

**Supplementary Figure 1A-1C**. Forest plots for the Mendelian randomization (MR) leave-one-out analysis of the significant inverse variance weighted (IVW) estimates. Within each panel, the black points represent the causal estimate of the association between a specific metabolite and epilepsy after discarding each SNP in turn. Red points represent the pooled IVW estimates. Horizontal lines denote 95% confidence intervals(*CI*). (A):biliverdin; (B):myristoleate; (C):1-palmitoylglycerophosphocholine.


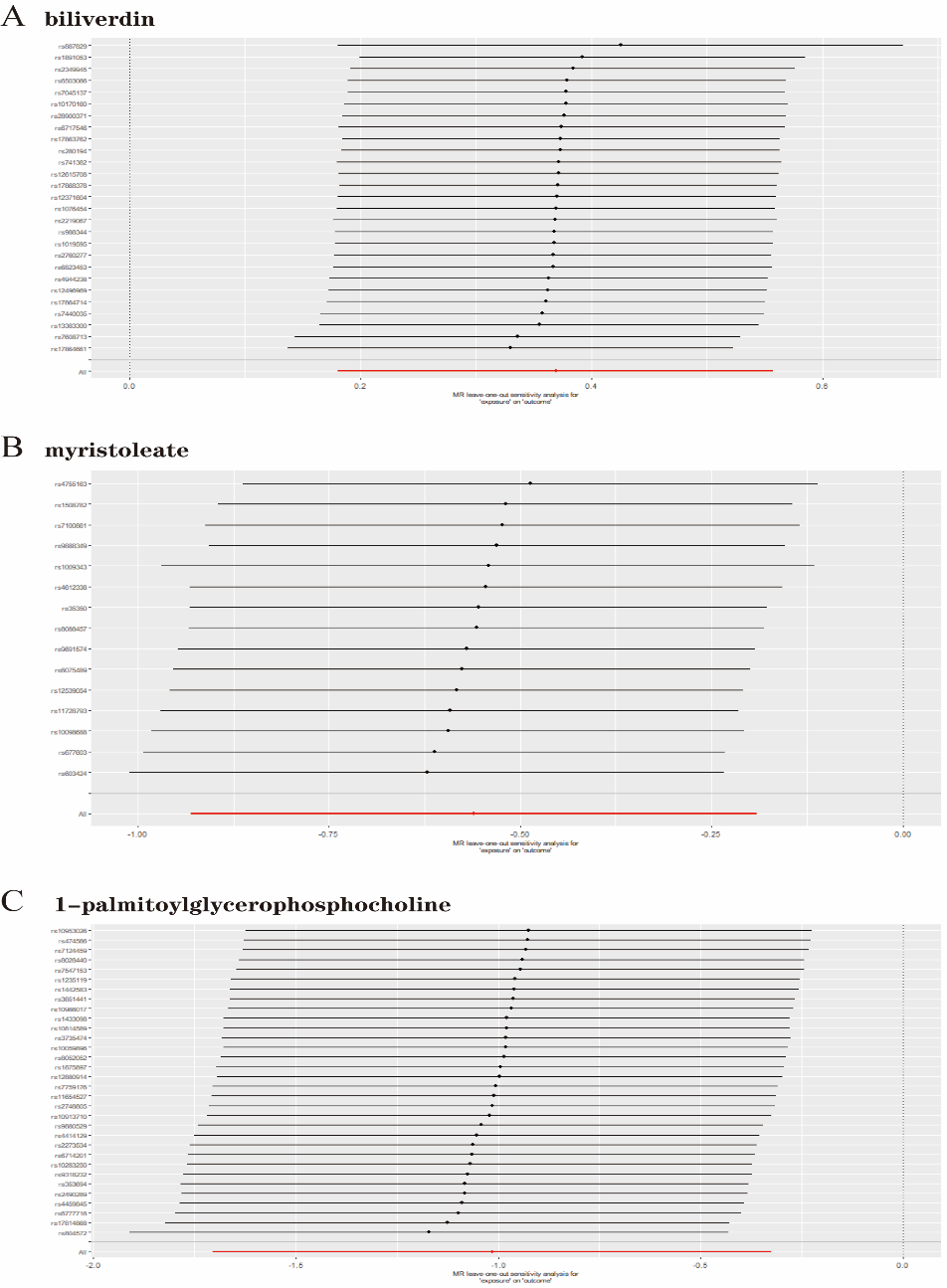

Supplement: Supplementary file 1 [file DataSheet1.docx]
